# Sphingosine-1-phosphate receptor 3 influences cell cycle progression in muscle satellite cells

**DOI:** 10.1016/j.ydbio.2013.07.006

**Published:** 2013-10-15

**Authors:** Mathieu Fortier, Nicolas Figeac, Robert B. White, Paul Knopp, Peter S. Zammit

**Affiliations:** King′s College London, Randall Division of Cell and Molecular Biophysics, New Hunt′s House, Guy′s Campus, London, SE1 1UL, UK

**Keywords:** Stem cell, Satellite cell, Skeletal muscle, Sphingosine-1-phosphate, S1PR3, Muscular dystrophy, *mdx*, Regeneration, Cell cycle, Proliferation, Quiescence

## Abstract

Skeletal muscle retains a resident stem cell population called satellite cells, which are mitotically quiescent in mature muscle, but can be activated to produce myoblast progeny for muscle homeostasis, hypertrophy and repair. We have previously shown that satellite cell activation is partially controlled by the bioactive phospholipid, sphingosine-1-phosphate, and that S1P biosynthesis is required for muscle regeneration. Here we investigate the role of sphingosine-1-phosphate receptor 3 (S1PR3) in regulating murine satellite cell function. *S1PR3* levels were high in quiescent myogenic cells before falling during entry into cell cycle. Retrovirally-mediated constitutive expression of *S1PR3* led to suppressed cell cycle progression in satellite cells, but did not overtly affect the myogenic program. Conversely, satellite cells isolated from *S1PR3*-null mice exhibited enhanced proliferation ex-vivo. In vivo, acute cardiotoxin-induced muscle regeneration was enhanced in *S1PR3*-null mice, with bigger muscle fibres compared to control mice. Importantly, genetically deleting *S1PR3* in the *mdx* mouse model of Duchenne muscular dystrophy produced a less severe muscle dystrophic phenotype, than when signalling though S1PR3 was operational. In conclusion, signalling though S1PR3 suppresses cell cycle progression to regulate function in muscle satellite cells.

## highlights

• Expression of S1PR3 is associated with non-cycling myoblasts.
• Constitutive expression of S1PR3 leads to reduced cell proliferation.
• Satellite cells lacking S1PR3 have enhanced proliferation.
• Muscle regeneration is improved in the absence of *S1PR3.*
• The dystrophic phenotype in *mdx* mice is improved by the absence of *S1PR3.*

## Introduction

Skeletal muscle retains a resident stem cell population called satellite cells, located on the myofibre surface (Mauro, 1961, Scharner and Zammit, 2011). In adult, these cells participate in muscle homeostasis, hypertrophy and repair. When satellite cells are genetically ablated though, skeletal muscle fails to regenerate (reviewed in Relaix and Zammit, 2012). Satellite cells are normally mitotically quiescent, but can be activated to enter the cell cycle and generate myoblast progeny. Most of these cells then differentiate to produce new myonuclei, while others self-renew to maintain the satellite cell pool (Collins et al., 2005, Zammit et al., 2004). While growth factors including HGF and FGFs are involved in the activation of satellite cells (Ten Broek et al., 2010), the mechanisms controlling activation and entry into cell cycle remain poorly understood.

Over the last few years the bioactive phospholipid, sphingosine-1-phosphate (S1P), has emerged as an important regulator of skeletal muscle function (Donati et al., 2013, Sabbadini et al., 1999). In Drosophila, perturbations in sphingosine levels lead to defects in muscle development and integrity (Herr et al., 2003), while elevation of intracellular S1P levels reduces muscle wasting in flies with dystrophic muscle (Pantoja et al., 2013). In mammals, S1P affects calcium homeostasis, cell contraction and differentiation of skeletal muscle (Donati et al., 2005, Formigli et al., 2002, Formigli et al., 2009). We have previously shown that S1P plays a crucial role in the entry of satellite cells into the cell cycle (Nagata et al., 2006a, Nagata et al., 2006b), and more recent work substantiates this pro-mitogenic effect of S1P on satellite cells (Calise et al., 2012, Loh et al., 2012). We also found that muscle regeneration is compromised when S1P biosynthesis is inhibited (Nagata et al., 2006b). Others have reported that S1P levels increase during muscle regeneration via both control of S1P biosynthesis and catabolism, and that muscle regeneration is augmented when exogenous S1P is administered (Danieli-Betto et al., 2010, Loh et al., 2012, Sassoli et al., 2011), confirming its central role in efficient muscle regeneration. The effects of S1P on myogenesis may not be restricted to satellite cells however, since S1P also influences proliferation and survival in mesoangioblasts, cells derived from the microvasculature with myogenic potential (Donati et al., 2007).

S1P can activate five cell surface G-protein-coupled receptors (S1PR1-5) that can operate through different signalling pathways (Rosen et al., 2009). S1PR1-4 are expressed in satellite cells, although their exact expression dynamics during myogenic progression are in debate (Calise et al., 2012, Danieli-Betto et al., 2010, Donati et al., 2005, Meacci et al., 1999, Pallafacchina et al., 2010). Ex-vivo, the mitogenic effects of S1P on satellite cells are mediated by S1PR2, and maybe S1PR3 (Calise et al., 2012), with S1P operating through at least S1PR2 in myogenic cells during muscle regeneration in vivo (Germinario et al., 2012, Loh et al., 2012). Finally, S1PR1 and S1PR3 appear to play antagonistic roles during muscle regeneration, where S1PR1 negatively and S1PR3 positively modulates the early phases of muscle regeneration (Danieli-Betto et al., 2010). Much of the work on the influence of S1PR on muscle regeneration has been conducted using small molecule antagonists and agonists. In particular, there are no specific antagonists or agonists for S1PR3, making it difficult to uncouple effects of perturbed S1PR3 signalling from those of non-S1PR3 effects (Pyne and Pyne, 2011, Salomone and Waeber, 2011).

Here we have characterized the role of the S1PR3 in skeletal muscle satellite cells using constitutive retroviral-mediated expression and knock-out mice to specifically examine S1PR3. We found that *S1PR3* expression is high in quiescent satellite cells and C2 reserve cells, compared to the levels in proliferating myoblasts. Since *S1PR3* expression is down-regulated in proliferating myoblasts, we used retroviral-mediated constitutive expression to examine the effects of maintaining high S1PR3 levels in proliferating satellite cell-derived myoblasts. This suppressed myoblast proliferation, but did not overtly affect myogenic progression. To investigate satellite cell function and muscle regeneration in the absence of signalling through S1PR3, we examined the *S1PR3*-null mouse (Ishii et al., 2001). Satellite cells isolated from *S1PR3*-null mice exhibited enhanced proliferative ability. In vivo, acute muscle regeneration following toxin-induced damage was enhanced in *S1PR3*-null mice. We also used the *mdx* mouse model of Duchenne muscular dystrophy to investigate if the absence of S1PR3 improved chronic muscle regeneration, and found that the dystrophic muscle phenotype was less severe in *S1PR3*^*−/−*^*/mdx* mice. Therefore, signalling through S1PR3 suppresses cell cycle progression and so plays a role in controlling satellite cell function.

## Materials and methods

### Animals

Maintenance and genotyping of *S1PR3*-null mice were as previously reported (Ishii et al., 2001). Heterozygotes were generated by breeding *S1PR3*-null mouse with C57 Bl/10 mice before inter-crossing to obtain wild-type, heterozygote and knock-out littermates and experiments performed using 6–10 week-old (25–35 g) males. *S1PR3*-null and *mdx* mice were crossed and female offspring were then crossed with a *S1PR3*-null male to obtain *mdx/S1PR3*^*+/−*^ and *mdx/S1PR3*^*−/−*^*-* progeny. Breeding and experimental procedures were passed by the Ethical Review Process Committee of King′s College London, and carried out under the provisions of the Animals (Scientific Procedures) Act 1986.

### Myofibre isolation

To obtain isolated myofibres, mice were killed by cervical dislocation and the extensor digitorum longus (EDL) muscle carefully dissected, and manipulated only by its tendons. EDL muscles were digested in 0.2% Collagenase Type 1 (Sigma, UK) in DMEM (Sigma, UK) supplemented with 400 mM l-Glutamine (Sigma, UK) and 1% (v/v) penicillin/streptomycin solution (Sigma, UK)] for 90 min at 37 °C. Individual myofibres were then dissociated by trituration using heat-polished glass Pasteur pipettes with variously sized apertures and washed, as described in detail elsewhere (Collins and Zammit, 2009, Rosenblatt et al., 1995).

### Preparation of quiescent satellite cells

To prepare quiescent satellite cells as near to mitotic quiescence as possible, freshly isolated myofibres were mildly digested with 0.125% trypsin-EDTA (Sigma, UK) for 12 min at 37 °C, before satellite cells were liberated using a heat-polished glass Pasteur pipette. Satellite cells were separated from myofibres and debris by first passing through a 40 μm cell sieve (BD Bioscience) followed by two rounds of centrifugation at 1000 rpm with PBS washes. Since little RNA can be obtained from quiescent satellite cells, myofibres from 4 to 6 EDL muscles were pooled for obtaining quiescent satellite cells for each replicate, and three replicates prepared (Knopp et al., 2013).

### Non-adherent myofibre culture

To study satellite cell-derived myoblasts while they remain retained on a myofibre, isolated myofibres were incubated in suspension in plating medium [DMEM with 10% (v/v) horse serum (PAA Laboratories, UK), 0.5% (v/v) chick embryo extract (ICN Flow), 400 mM l-glutamine (Sigma, UK) and 1% (v/v) penicillin/streptomycin solution (Sigma, UK)] in 50 mm×18 mm non-tissue culture petri dishes (Sterilin 124) coated with 0.1% BSA/PBS at 37 °C in 5% CO_2_.

### Preparation of satellite cell-derived primary myoblasts

For adherent cultures, isolated myofibres were plated in 6-well plates (Nunc, UK) coated with 1 mg/ml Matrigel (Collaborative Research). Plating medium was added and the cultures maintained at 37 °C in 5% CO_2_. After 72 h in culture, myofibres were removed, and the remaining satellite cell-derived myoblasts trypsinised and re-plated in Matrigel-coated LAB-TEK 8-well chamber slides (Nunc, UK) and expanded using growth medium [DMEM supplemented with 30% (v/v) foetal calf serum, 10% (v/v) horse serum, 1% (v/v) chick embryo extract, 10 ng/ml bFGF, 400 mM l-glutamine (Sigma, UK) and 1% (v/v) penicillin/streptomycin solution (Sigma)]. For EdU experiments, bFGF was omitted from the proliferation medium. To induce differentiation, myoblasts were cultured in DMEMGlutamax (Invitrogen) with v/v 2% horse serum (Gibco) and 1% (v/v) penicillin/streptomycin solution (Sigma, UK)].

### Quantitative RT-PCR

Total RNA was extracted using the RNeasy Kit (Qiagen, UK) and cDNA prepared from 100 to 500 ng of RNA with the QuantiTect Reverse Transcription Kit with genomic DNA wipeout (Qiagen, UK). QPCR was performed on an Mx3005P QPCR system (Stratagene, UK) with Brilliant II SYBR green reagents and ROX reference dye (Stratagene, UK). Primers used in this study were: *s1p1* (forward 5′-TCATAGTCCGGCATTACAACTA-3′, reverse 5′-GTGTGAGCTTGTAAGTGGTG-3′), *s1p2* (forward -GCAGTGACAAAAGCTGCCGAATGCTGATG-3′; reverse 5′AGATGGTGACCACGCAGAGCACGTAGTG-3′), *s1p3* (forward 5′ TCAGTATCTTCACCGCCATT-3′; reverse 5′-AATCACTACGGTCCGCAGAA-3′), *Gapdh* (forward 5′ GTGAAGGTCGGTGTGAACG 3′, reverse 5′ ATTTGATGTTAGTGGGGTCTCG 3′), *p27 (Cdkn1b)* (forward 5′GTGGACCAAATGCCTGACTC 3′, reverse 5′ TCTTCTGTTCTGTTGGCCCT 3′), *myogenin* (forward 5′ CTACAGGCCTTGCTCAGCTC 3′, reverse 5′ AGATTGTGGGCGTCTGTAGG 3′).

### Retroviral expression vectors

The retroviral backbone *pMSCV-puro* (Clontech) was modified to replace the puromycin selection gene with eGFP, to create *pMSCV-IRES-eGFP*, which served as the control vector (Zammit et al., 2006). Murine S1PR cDNAs (S1PR1 NM_007901.5; S1PR2 NM_010333.4; S1PR3 NM_010101.4) were individually cloned into *pMSCV-IRES-eGFP* to generate *pMSCV-S1PR1-IRESeGFP*, *pMSCV-S1PR2-IRESeGFP* or *pMSCV-S1PR3-IRESeGFP,* producing each receptor as a bicistronic message with *eGFP*. Constructs were sequenced and verified by qPCR. Retroviral constructs, together with an ecotropic packaging plasmid, were transiently co-transfected into 293T cells to produce non-replicating retrovirus and the supernatant harvested.

### Retroviral infection

A total of 5000 primary myoblasts were plated in each well of LAB-TEK 8-well chamber slides (Nunc, UK). After 24 to 48 h, the medium was replaced with a 1:5 dilution of 293T retroviral supernatant with 4 mg/ml polybrene and incubated at 37 °C for 6 h, before cells were rinsed and placed in fresh medium. To infect satellite cells associated with myofibres, they were exposed to a 1:10 dilution of the supernatant after 24 h in culture, and the cells were then analysed 48 h later.

### Immunocytochemistry

Primary antibodies used were: mouse monoclonal anti-Pax7 and anti-myogenin clone F5D (Developmental Studies Hybridoma Bank, Iowa, USA); mouse monoclonal anti-MyoD clone 5.8A (DakoCytomation); rabbit polyclonal anti-MyoD (Santa Cruz Biotechnology); polyclonal rabbit anti-GFP (Invitrogen); rat monoclonal anti-Ki67 (Dako Ltd., Kyoto, Japan). Immunostaining was performed as described in detail elsewhere (Beauchamp et al., 2000). Species specific fluorochrome-conjugated secondary antibodies (Invitrogen) were then applied for 1–2 h, before mounting in Vectashield mounting medium with DAPI (Vector Laboratories, Inc.). The Click-iT EdU imaging kit (Invitrogen, LifeTechnologies, Paisley, UK) was used as per the manufacturer′s instructions. Immunostained myofibres, plated cells or cryosections were viewed on a Zeiss Axiophot 200M using Plan-Neofluar lenses (Zeiss, WelwynGarden City, Hertfordshire, UK), or on a Nikon (Kingston upon Thames, Surrey, UK) C1si confocal using Plan-Fluor lenses. Digital images were acquired with a ZeissAxioCam HRm Charge-Coupled Device using AxioVision software version 4.4.

### Muscle regeneration

The left and right TA muscles of 6–8 week-old male heterozygote or *S1PR3*-null mice were injected with 25 µl of 10 µM cardiotoxin (Sigma). We used two injection regimes: either a single cardiotoxin injection, with muscle analysed after 7 and 21 days, or three injections at one week intervals, with muscles assayed 7 days after the final injection (21 days). Muscles were immediately frozen in isopentane cooled in liquid nitrogen and stored at −80 °C. Transverse muscle sections were cut with a cryostat and stained with haematoxylin and eosin to examine any gross morphological differences. Quantification of centrally nucleated regenerating myofibres and measurement of myofibre cross sectional area were performed using ImageJ software (developed by NIH and available for free download at http://rsb.info.nih.gov/ij).

### Statistical analysis

Each experiment was repeated independently at least three times (*n*=3–6 depending on the experiment) and the mean from multiple samples determined from each animal, and the population mean±SEM calculated. Differences between test conditions and controls were subjected to statistical analysis using *T*-test in the Microsoft Excel Software (Microsoft Excel Corp., New York, USA). Statistical significance was accepted at *p*<0.05. Details of numbers and statistical testing are given in figure legends.

## Results

### S1PR3 is expressed at a high level in quiescent myogenic cells

Higher *S1PR3* expression in quiescent versus proliferating satellite cells has been reported using microarray analysis (Pallafacchina et al., 2010), and immunostaining with a non-commercially available antibody has shown S1PR3 on quiescent satellite cells (Danieli-Betto et al., 2010). We first examined the expression profile of *S1PR1, S1PR2* and *S1PR3* in myogenic cells in detail using the reserve cell model of induced myogenic quiescence (Yoshida et al., 1998). Proliferating C2C12 myoblasts were induced to differentiate by serum withdrawal and 7 days later, the reserve cells were separated from myotubes by differential trypsinization, and gene expression levels measured by QPCR. Compared to the levels in proliferating C2 cells, we found that *S1PR1* was slightly increased in myotubes while *S1PR2* levels fell, with *S1PR3* remaining unchanged. By contrast in quiescent reserve cells, while *S1PR2* was increased 6.5 fold, *S1PR3* levels were ~43 fold higher (Fig. 1A). The cell cycle inhibitor *p27 (Cdkn1b)* was also significantly increased in both myotubes and reserve cells, confirming exit from the cell cycle. Stimulating reserve cells with high-serum medium results in their rapid re-entry into the cell cycle, as shown by the drop in *p27 (Cdkn1b)* levels by 2 h. Measuring gene expression at 2 h intervals revealed that *S1PR3* levels also fell dramatically, becoming indistinguishable from the amount in proliferating cells by 6 h after stimulation (Fig. 1A). Even by 6 h though, *S1PR1* and *S1PR2* levels had not returned to those found in proliferating cells (Fig. 1A).

**Fig. 1:**
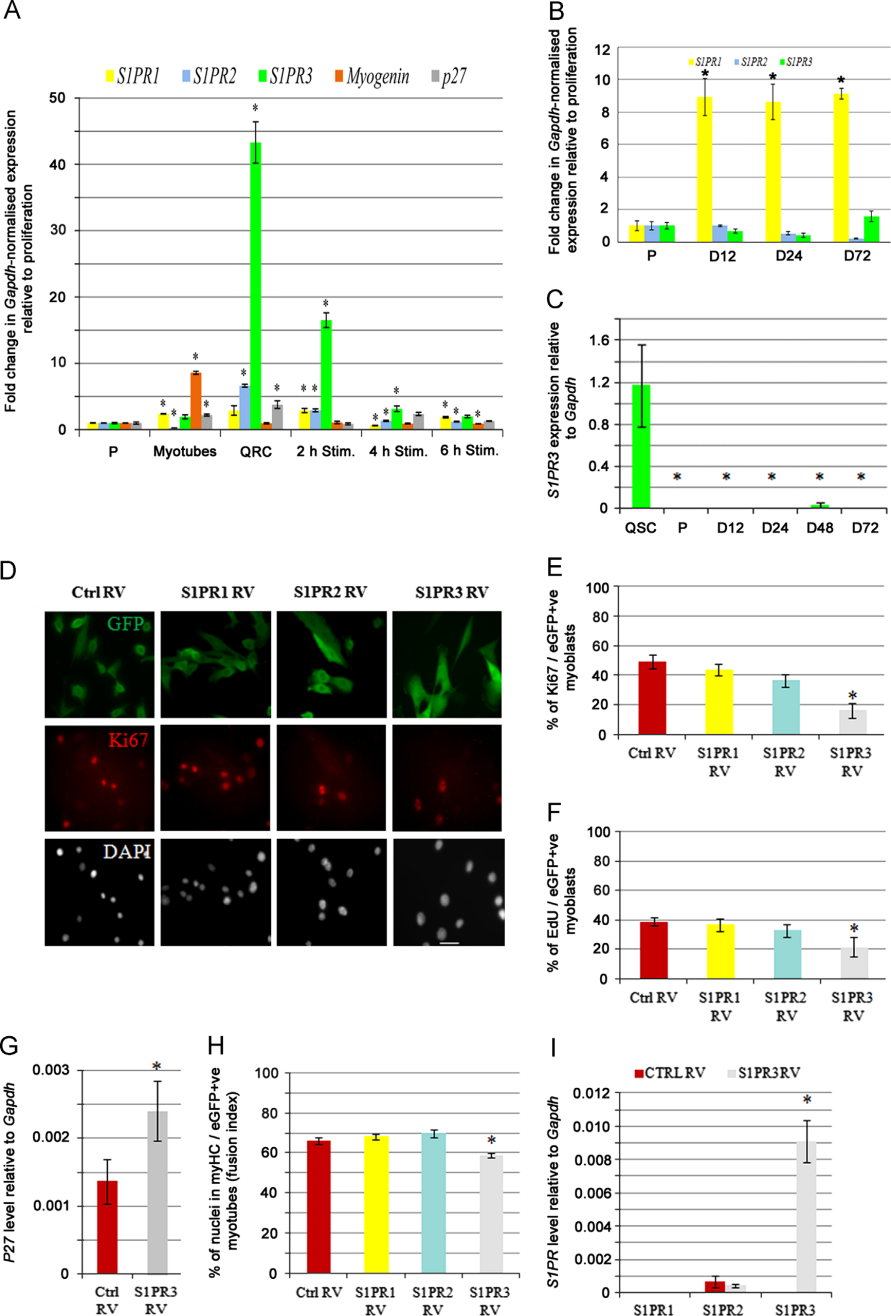
S1PR3 suppresses cell cycle progression in satellite cells (A) RNA was prepared from either proliferating (P), differentiated (myotubes) or quiescent reserve C2 cells (QRC). After removal of myotubes, quiescent reserve cells were then stimulated to re-enter the cell cycle by exposure to high-serum medium, and RNA prepared at 2 h intervals for 6 h (2 h Stim., 4 h Stim. and 6 h Stim.). Levels of *S1PR* mRNA was analysed by QPCR, with *Myogenin* and *p27 (Cdkn1b)* used as internal controls for differentiation and exit from cell cycle. *S1PR3* was increased ~43 fold in quiescent reserve cells compared to levels in proliferating C2 cells. *S1PR3* levels then fell as the reserve cells were stimulated to re-enter the cell cycle, dropping to levels equivalent to those in proliferating C2 cells by 6 h. (B) RNA was also prepared from plated proliferating satellite cell-derived myoblasts (P), and after 12 (D12), 24 (D24) and 72 h (D72) in differentiation medium. Levels of *S1PR1* rose during differentiation, while those of *S1PR2* and *S1PR3* were unchanged. (A) and (B) Values are mean±SEM fold change from levels in proliferating cells from three separate experiments or mice (*n*=3), where an asterisk denotes significant difference *(p*<0.05) in gene expression from the level in proliferating cells using a two-tailed *T*-test. (C) *S1PR3* expression was measured in RNA from satellite cells stripped from freshly isolated EDL myofibres (QSC) and compared to the levels in plated proliferating (P) and differentiating (D12–D72) satellite cell-derived myoblasts. *S1PR3* was at significantly higher levels in quiescent, versus proliferating or differentiating cells (mean±SEM from 3 mice, where an asterisk denotes significant difference from the levels in quiescent cells [*p*<0.05] using a one-tailed *T*-test). (D)–(F) To determine the effects of constitutive expression of S1PR, plated expanded satellite cells were infected with retroviruses encoding either S1PR1 (S1PR1 RV), S1PR2 (S1PR2 RV) or S1PR3 (S1PR3 RV) as a bicistronic message with *IRES-eGFP*, or control RV, and cultured for a further 48 h before fixation. (D) Cells were co-immunostained for eGFP (green) to identify infected cells and Ki67 (red) to determine whether the cell was in the cell cycle, together with DAPI counterstaining to identify all cells present. (E) Constitutive S1PR3 expression resulted in significantly fewer cells in the cell cycle compared to Ctrl RV infection, while neither S1PR1 nor S1PR2 affected the proportion of proliferating cells. (F) Infected proliferating satellite cell-derived myoblasts were also pulsed with EdU for 2 h before fixation. Quantification of immunostaining for eGFP and EdU revealed a significant decrease in the proliferation rate of myoblasts with constitutive *S1PR3* expression, but not with *S1PR1* or *S1PR2*, compared to control. (G) QPCR analysis of the relative expression levels of *p27 (Cdkn1b)* in infected satellite cells showed that cells with constitutive *S1PR3* expression had higher expression of *p27 (Cdkn1b)* than controls. (H) To examine differentiation in the presence of constitutive S1PR1, S1PR2 or S1PR3, plated expanded satellite cells were infected and cultured for a further 2 days before switching to differentiation medium for 2 days and fixed. Quantification of nuclei in myotubes co-immunostaining for eGFP and MyHC showed a small decrease in the fusion index in S1PR3 RV-infected satellite cell-derived myoblasts. (I) QPCR analysis of S1PR3 RV-infected satellite cells showed that only *S1PR3* expression showed a significant change (increase) relative to control RV, with *S1PR1* and *S1PR2* levels unaffected by constitutive *S1PR3* expression. Values in (D)–(I) are mean±SEM from three mice (*n*=3), where an asterisk denotes significantly different from control infection, with *p*<0.05 using a two-tailed *T*-test.

We then determined the dynamics of *S1PR* expression in primary murine satellite cells during myogenic progression. Myofibres from mouse EDL muscles were isolated by collagenase digestion, and the muscle fibres plated on Matrigel-coated tissue culture dishes. Once satellite cell-derived myoblasts had accumulated around myofibres, the muscle fibres were removed, and the cells passaged, pooled and re-plated. RNA was prepared from proliferating cells, and after 12, 24 and 72 h in differentiation medium (Fig. 1B). Compared to the levels in proliferating satellite cell-derived myoblasts, the levels of *S1PR1* rose significantly during myogenic differentiation, while *S1PR2* and *S1PR3* expression was unaltered (Fig. 1B).

Since *S1PR3* in particular, is expressed at much higher levels in C2 reserve cells compared to those in proliferation, we attempted to determine if this was also the case for satellite cells. To obtain RNA from satellite cells as near to mitotic quiescence as possible, cells were stripped from freshly isolated EDL myofibres from several mice and pooled. *S1PR3* levels were measured by QPCR in parallel with proliferating satellite cell-derived myoblasts and during myogenic differentiation. Expression of *S1PR3* was significantly higher in quiescent satellite cells compared to either proliferating or differentiating satellite cells (Fig. 1C). Thus *S1PR3* levels are higher in quiescent myogenic cells than those in the cell cycle.

### Constitutive S1PR3 expression suppresses cell cycle progression

Since *S1PR3* is normally down-regulated in proliferating myogenic cells, we set out to determine the effects of maintaining high *S1PR3* expression throughout the cell cycle. Retroviruses were generated to express *S1PR1 (S1PR1 RV)*, *S1PR2 (S1PR2 RV)* or *S1PR3 (S1PR3 RV)*, together with an IRES-controlled eGFP. Plated, expanded proliferating satellite cell-derived myoblasts from the EDL were infected with either *S1PR*-expressing- or control retrovirus (containing no insert), and cultured for 48 h, before being fixed and immunostained for the cell cycle marker Ki67 and eGFP. Satellite cells over-expressing *S1PR1* or *S1PR2* (eGFP+ve) behaved as those infected with control retrovirus, with ~50% of cells in the cell cycle as shown by nuclear Ki67. By contrast, only ~18% of infected eGFP-expressing satellite cells with retroviral-mediated expression of *S1PR3* contained nuclear Ki67 (Fig. 1D and E), showing that S1PR3 promotes cell cycle exit. We also pulsed infected proliferating satellite cells with EdU for 2 h and examined the level of incorporation. Significantly fewer cells over-expressing *S1PR3* (eGFP+ve) contained EdU, compared to those expressing control retrovirus: revealing a decreased proliferation rate in satellite cells with retroviral-mediated expression of *S1PR3* (Fig. 1F). The proliferation rate of cells expressing *SIPR1* or *S1PR2* was unchanged from controls (Fig 1F). Consistent with these observations, retroviral-mediated *S1PR3* expression in satellite cells significantly increased levels of the cell cycle inhibitor *p27 (Cdkn1b)* (Fig. 1G).

We next investigated if retroviral-mediated expression of *S1PR1*, *S1PR2* or *S1PR3* affected myogenic differentiation. Satellite cells were infected with S1PR1 RV, S1PR2 RV, S1PR3 RV or control RV and then cultured for a further 2 days, before being switched to differentiation medium for 2 days before fixation, by which time there was extensive fusion into multinucleated myotubes. Co-immunostaining for myosin heavy chain (MyHC) and eGFP was performed and the percentage of nuclei within MyHC/eGFP+ve myotubes (defined as having 2 or more nuclei) determined to calculate the fusion index. This revealed that there was a small, but significant, decrease in myogenic differentiation following retroviral-mediated expression of *S1PR3* (Fig. 1H). Despite the increased expression of *S1PR1* in differentiating myoblasts, constitutive expression of *S1PR1* did not further enhance myoblast fusion (Fig. 1H).

To determine if the decrease in proliferation in cells over-expressing *S1PR3* leads to precocious myogenic differentiation, satellite cell-derived myoblasts were infected with S1PR3 RV and control RV and after 24 h, given a 2 h EdU pulse and fixed. Sister cultures were then co-immunostained for either Pax7/eGFP or myogenin/eGFP, combined with EdU detection. Again, there were fewer eGFP+ve cells incorporating EdU after infection with *S1PR3* RV than control RV, but there was no change in the proportion of eGFP+ve cells expressing Pax7 (70.5±2.1 for S1PR3 RV compared to 72.9±1.2 for control RV) or myogenin (23.3±1.2 for *S1PR3* RV compared to 21.3±2.1 for control RV).

To examine if increased S1PR3 levels in satellite cells due to retroviral infection affected other S1PR, we measured *S1PR1* and *S1PR2* by QPCR. Neither *S1PR1* nor *S1PR2* were significantly changed by retroviral-mediated constitutive *S1PR3* expression, while *S1PR3* levels were significantly elevated (Fig. 1I).

### S1PR3-null satellite cells have enhanced proliferation

We next examined the effects of an absence of signalling through S1PR3 using *S1PR3*-null mice. While *S1PR3*-null mice have been reported to have a reduced litter size, surviving mice do not have an overt phenotype (Ishii et al., 2001), so provide a useful model to examine the effects of S1PR3 on satellite cell function. We first examined by Q-PCR, whether the lack of signalling through S1PR3 in *S1PR3*-null mice affected expression of *S1PR1* or *S1PR2* in muscle and myogenic cells (Supplementary Fig. 1)*. S1PR1* levels were unaltered in RNA from either whole EDL muscle or proliferating EDL satellite cell-derived myoblasts, while *S1PR2* expression was increased in RNA from whole EDL muscle (Supplementary Fig. 1A) but not from proliferating myoblasts (Supplementary Fig. 1B).

**Fig. 2:**
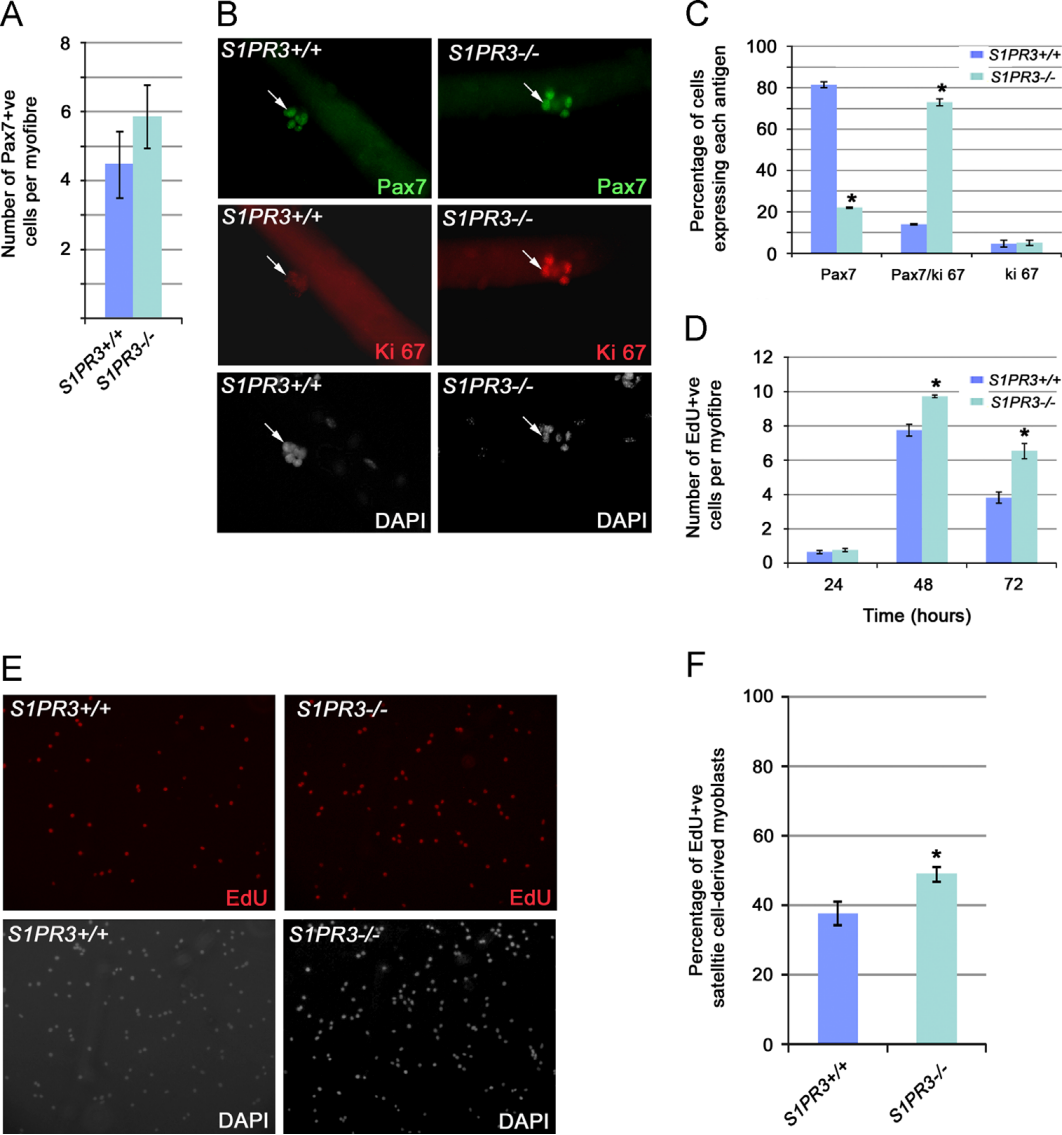
*S1PR3*-null satellite cells have increased proliferation. (A) EDL myofibres were isolated from age-matched wild-type or *S1PR3*-null mice and immunostained for Pax7 to identify quiescent satellite cells. There was no difference in the number of quiescent satellite cells per EDL myofibre in 6–8 week old *S1PR3-*null or wildtype mice. (B) and (C) Satellite cells associated with a myofibre from *S1PR3*^*−/−*^ and control mice were cultured for 72 h and co-immunostained for Pax7 (green) and Ki67 (red), with DAPI counterstaining, to assess proliferation and self-renewal. A significantly lower proportion of satellite cells lacking S1PR3 exited the cell cycle to adopt the self-renewal phenotype compared to wild type cells. (D) Satellite cells associated with a myofibre from *S1PR3*^*−/−*^ and control mice were cultured in medium to promote satellite cell proliferation and samples pulsed with EdU for 4 h and fixed at 24 h intervals. More *S1PR3*-null satellite cells had incorporated EdU after 48 and 72 h, compared to controls. (E) and (F) Plated expanded satellite cell-derived myoblasts were cultured in serum-rich medium and pulsed for 2 h with EdU before fixation. Co-staining for EdU and DAPI confirmed that more satellite cells from *S1PR3*-null mice incorporated EdU compared to control cells. Values are mean±SEM from three mice (*n*=3) where an asterisk denotes significantly different from wild-type controls (*p*<0.05) using a two-tailed *T*-test.

Immunostaining for Pax7 on freshly isolated intact EDL myofibres permits the number of quiescent satellite cells to be counted. We found that there was no significant difference in the number of quiescent satellite cells per myofibre between adult (6–8 weeks of age) *S1PR3*-null and control wild-type mice (Fig. 2A), indicating that loss of S1PR3 does not affect the generation of satellite cells during development.

To address proliferation and self-renewal of satellite cells in the absence of S1PR3, we cultured EDL myofibres and their associated satellite cells from *S1PR3*-null and wildtype control mice for 72 h, before fixation and co-immunostaining for Ki67 and Pax7 (Fig. 2B and C). By 72 h, many satellite cells are adopting a phenotype consistent with self-renewal, i.e. express Pax7 and are no longer proliferating (Zammit et al., 2004). There was a significantly lower proportion of Pax7+/Ki67-ve satellite cells (considered to be undergoing self-renewal) in *S1PR3*-null mice compared to controls (~20% versus ~80%, respectively—Fig. 2B and C). Thus at a time when many satellite cells are no longer proliferating in wildtype mice, there are many still in the cell cycle when *S1PR3* is genetically inactivated.

To assess the proliferation rate of *S1PR3-*null satellite cells, myofibre-associated satellite cells were cultured, and samples pulsed with EdU and fixed at 24 h intervals. Myofibres were co-immunostained for MyoD and EdU and the number of cells per myofibre that had incorporated EdU counted. There was a significant increase in the number of EdU-containing satellite cells from *S1PR3*-null mice after 48 and 72 h of culture, compared to wildtype controls (Fig. 2D).

We also assessed proliferation in plated satellite cell-derived myoblasts, as proliferation on myofibres is limited by the entry of cells into self-renewal or differentiation by approximately 72 h. Satellite cells from *S1PR3*-null and wildtype mice were pulsed with EdU for 2 h and then fixed. A significantly higher proportion of satellite cells from *S1PR3*^*−/−*^ mice incorporated EdU, when compared to cells isolated from controls (Fig. 2E, quantified in Fig. 2F).

### Myogenic progression in S1PR3-null satellite cells is relatively unaffected

**Fig. 3:**
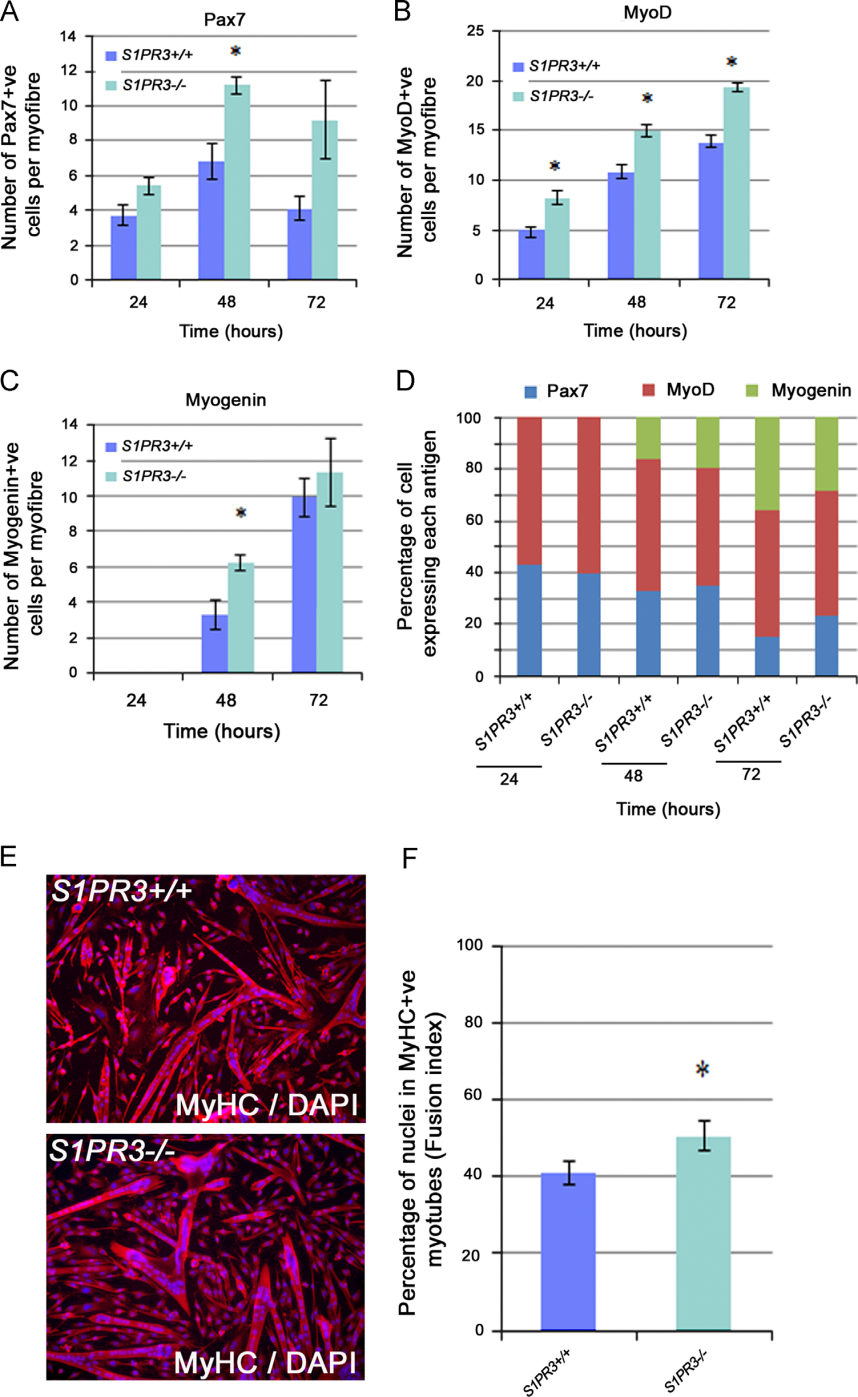
Myogenic progression in *S1PR3*-null satellite cells is relatively normal. EDL myofibres and their associated satellite cells were isolated from either age-matched wild-type (*S1PR3*^*+/+*^) or *S1PR3-*null (*S1PR3*^*−/−*^) mice and cultured in proliferation medium for 72 h. (A)–(C) Samples were taken at 24 h intervals, fixed and co-immunostained for Pax7, MyoD and Myogenin, which showed a general increase in each population when S1PR3 was absent, consistent with enhanced proliferation. (D) When expressed as a ratio though, the proportions of cells expressing Pax7, MyoD or myogenin were similar between wildtype and *S1PR3*-null mice. (E) and (F) Expanded satellite cell-derived myoblasts were also plated at high confluency and then cultured in differentiation medium for 48 h before immunostaining for Myosin Heavy Chain (MyHC) and counterstaining with DAPI. Counting the number of nuclei within MyHC+ve myotubes (>2 nuclei) to determine the fusion index revealed that more satellite cells from *S1PR3*-null mice had differentiated and fused than from control mice ((E), quantified (F)). Values are expressed as mean±SEM from multiple samples from at least three mice (*n*=3/4) where an asterisk denotes significantly different (*p*<0.05) from wild-type controls using a two-tailed *T*-test.

Having found that a lack of S1PR3 enhances proliferation in satellite cells, we then investigated whether it also affected the myogenic program. EDL-derived myofibres with their associated satellite cells were cultured for 72 h, with samples fixed at 24 h intervals and co-immunostained for Pax7, MyoD or myogenin (Zammit et al., 2004). There was a general increase in the number of satellite cell-derived myoblast progeny (Fig. 3A–C), consistent with the enhanced proliferation detected by the Ki67 and EdU pulsing experiments (Fig. 2). Importantly, the proportion of satellite cells expressing Pax7, MyoD and Myogenin was similar between wild type and *S1PR3*^*−/−*^ derived satellite cells (Fig. 3D), reinforcing the idea that an increase in proliferation, rather than any overt modification in myogenic progression, is the main affect of the absence of S1PR3.

To examine differentiation into multi-nucleated myotubes, expanded satellite cell-derived myoblasts were plated at high confluence and immediately switched to differentiation medium, to limit any effects of differential proliferation rates. Two days later, cells were fixed and immunostained for Myosin Heavy Chain and counterstained with DAPI. Counting the number of nuclei within MyHC+ve myotubes (>2 nuclei) to determine the fusion index revealed that more satellite cells from *S1PR3*-null mice had differentiated and fused than from control mice (Fig. 3E, quantified Fig. 3F). Thus, lack of S1PR3 increases proliferation and improves myogenic fusion, without drastically affecting myogenic progression.

### Acute muscle regeneration is improved in S1PR3-null mice

**Fig. 4:**
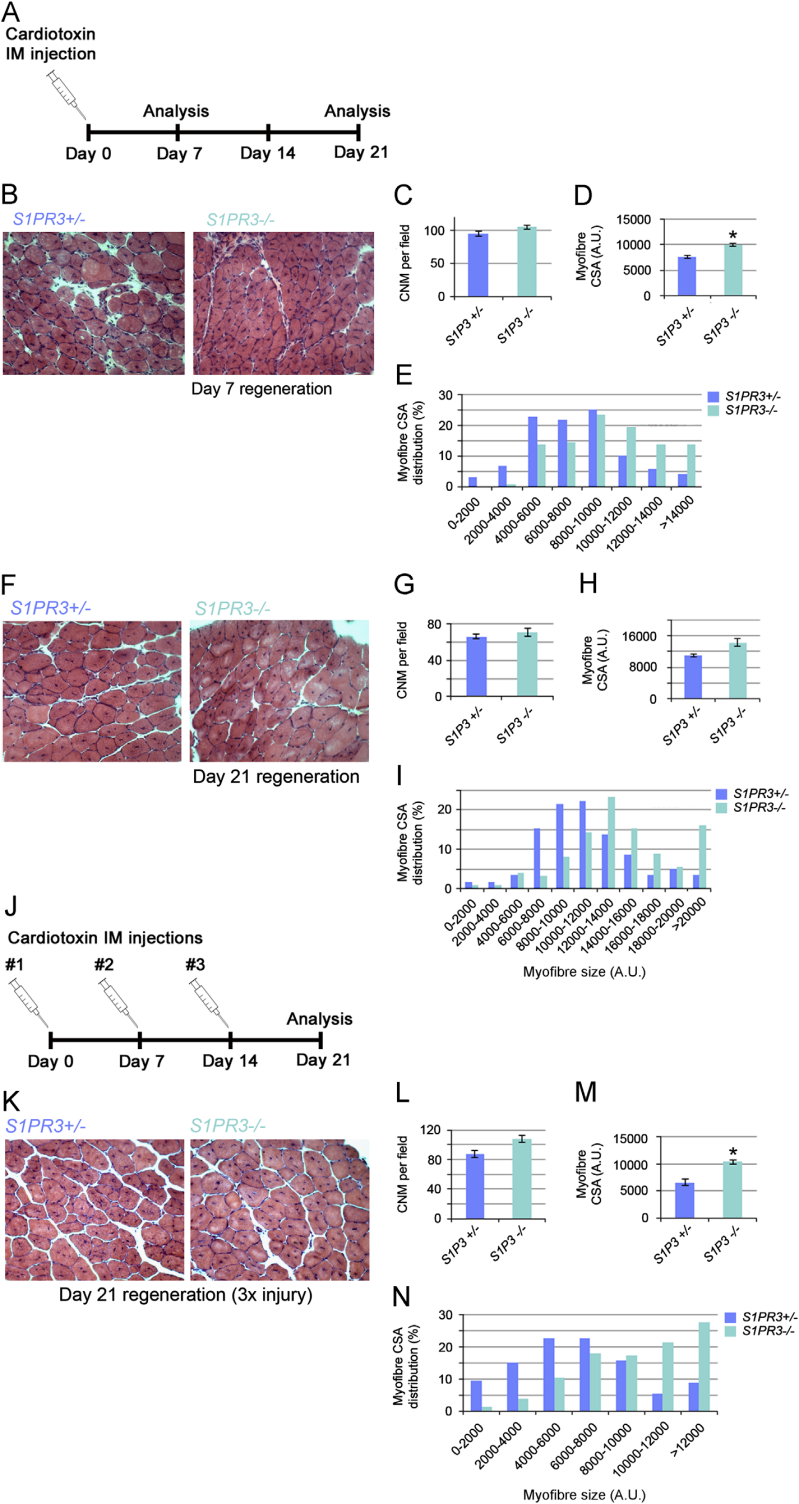
Absence of S1PR3 enhances acute muscle regeneration. (A) Both Tibialis Anterior muscles from either age-matched *S1PR3*^*+/−*^ or *S1PR3*^*−/−*^ mice were injected with cardiotoxin, and removed either 7 or 21 days later, cryosectioned and stained with haematoxylin and eosin. (B)–(C) The presence of myofibres with centrally-located myonuclei (CNM) showed that skeletal muscle regenerated successfully in the absence of S1PR3. (D) - (E) Myofibres had a significantly larger mean cross-sectional area (CSA) in regenerating TA of *S1PR3*-null mice compared to *S1PR3*^*+/−*^ controls, with a bias towards larger size. (F)–(I) After 21 days from the time of injury, there was no longer any difference in the mean size of myofibres. (J) Mice were also subjected to a more rigorous acute regeneration regime of three consecutive rounds of cardiotoxin-induced injury 7 days apart. (K)–(L) Haematoxylin and eosin staining 7 days after the third injury revealed many centrally-nucleated myofibres present, showing that *S1PR3*^*−/−*^ mice still regenerated effectively after this more challenging injury routine. (M) - (N) Myofibres in regenerated muscle from *S1PR3*-null mice had a higher mean cross sectional area than in control heterozygotes with a bias towards a larger size. Values are mean±SEM of a minimum of 40 myofibres from each of 9–13 fields from multiple sections per animal for determining the number of myofibres with centronucleation per unit area, or from between 32 and 68 centrally-nucleated myofibres from a single representative field per animal for measuring CSA, each from 3 mice per genotype. For D, H and M, an asterisk denotes where *S1PR3*^*−/−*^ are significantly different (*p*<0.05) from *S1PR3*^*+/−*^ controls using a two-tailed *T*-test.

To assess satellite cell function in the absence of S1PR3 in vivo, muscle regeneration following myotoxin-induced damage was analysed in *S1PR3*^*−/−*^ and control *S1PR3*^*+/−*^ mice. A single intramuscular injection of cardiotoxin was administered to each Tibialis Anterior (TA) of *S1PR3*-null or control heterozygote mice and the muscles analysed after 7 or 21 days of regeneration (Fig. 4A). Cryosections were prepared for haematoxylin and eosin staining to examine gross morphology. Skeletal muscle was able to robustly regenerate in mice lacking S1PR3, as shown by the presence of many myofibres with centrally-located nuclei (a hallmark of myofibre regeneration) (Fig. 4B, C, F and G). After 7 days of regeneration, there was a significant increase in the mean myofibre size in *S1PR3*^*−/−*^ compared to *S1PR3*^*+/−*^ mice, as determined by measuring cross-sectional area (Fig. 4B, quantified in Fig. 4D and E). By 21 days after the initiation of regeneration though, there was no longer any difference in myofibre size in TA muscles of *S1PR3*-null compared to control *S1PR3*^*+/−*^ mice (Fig. 4F, quantified in Fig. 4H and I).

We also subjected the TA to a more rigorous regime of repeated acute regeneration with three intramuscular injections of cardiotoxin at one-week intervals, with analysis 7 days after the final injection (21 days) (Fig. 4J). There was still a clear regenerative ability after this more challenging regime of multiple rounds of muscle damage, with *S1PR3*-null mice having many centrally nucleated myofibres (Fig. 4K and L). There was a significant increase in the mean cross-sectional area of muscle fibres in the *S1PR3*-null mice after the muscle injury/recovery program, with more large myofibres present, indicating enhanced regeneration (Fig. 4M and N).

### Absence of S1PR3 improves the dystrophic muscle phenotype in mdx mice

To also examine the effects of a lack of S1PR3 signalling on chronic muscle regeneration, we examined the dystrophic phenotype in the *mdx* mouse model of Duchenne muscular dystrophy. The *mdx* mouse undergoes chronic rounds of myofibre degeneration/regeneration from approximately 3 weeks of age (De la Porte et al., 1999). *S1PR3*-null and *mdx* mice were crossed to generate *mdx* male mice that were either heterozygous for *S1PR3* (control *mdx*/*S1PR3*^*+/−*^) or *S1PR3*-null (*mdx/S1PR3*^*−/−*^).

**Fig. 5:**
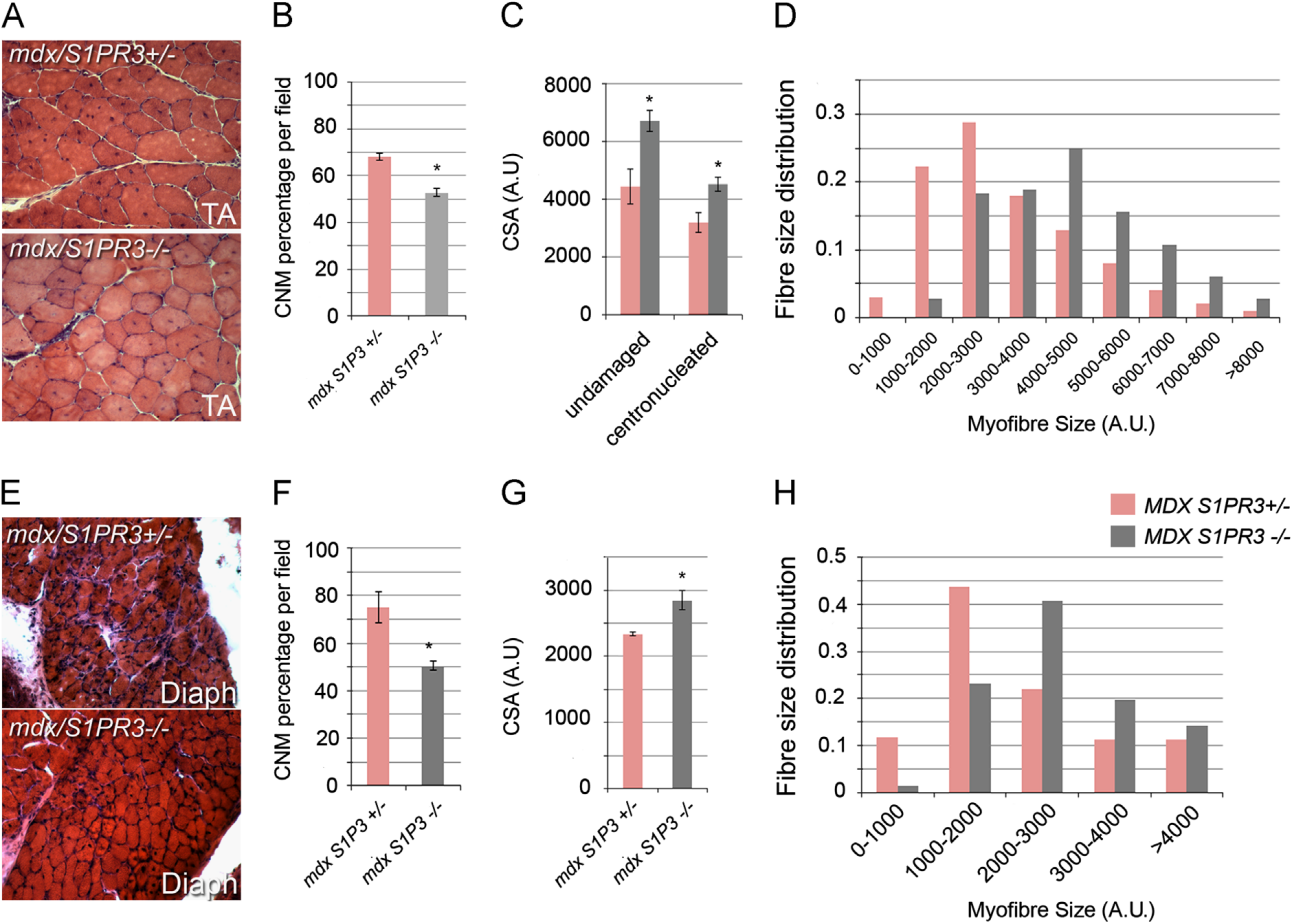
Absence of S1PR3 improves the dystrophic muscle phenotype in *mdx* mice. Tibialis Anterior (TA) and Diaphragm (Diaph) from separate age-matched control *mdx/S1PR3*^*+/−*^ or *mdx/S1PR3*^*−/−*^ males were cryosectioned and stained with haematoxylin and eosin. (A)–(B) Tibialis Anterior muscle of *mdx/S1PR3*^*−/−*^ had a significant decrease in the number of centrally nucleated myofibres (CNM) per unit area compared to control *mdx/S1PR3*^*+/−*^ mice. (C) Mean cross-sectional area (CSA) of myofibres with either peripherally-located and centrally-located myonuclei was also increased in *mdx/S1PR3*^*−/−*^ compared to control *mdx/S1PR3*^*+/−*^ mice, (D) with a bias towards larger sized centrally-nucleated myofibres. (E) to (F) Diaphragm from *mdx/S1PR3*^*−/−*^ had significantly fewer centrally nucleated myofibres (CNM) compared to control *mdx/S1PR3*^*+/−*^ littermates. (G) - (H) Cross-sectional area (CSA) of myofibres per unit area was increased in *mdx/S1PR3*^*−/−*^ compared to control *mdx/S1PR3*^+/*−*^ mice, with a predominance of larger myofibres. Values are mean±SEM of between 300 and 492 myofibres from each of 2–4 fields from multiple sections per animal from 5 to 6 mice per genotype for determining the degree of centronucleation in TA muscles, and of between 120 and 320 myofibres from 3 to 8 fields from multiple sections per animal from 3 mice per genotype for assessing centronucleation in diaphragm. CSA is mean±SEM from measuring between 31 and 76 myofibres from a single representative field per muscle per animal from 3 mice per genotype. An asterisk denotes where *mdx/S1PR3*^*−/−*^ are significantly different (*p*<0.05) from *mdx/S1PR3*^*−/+*^ controls using two tailed *T*-test.

TA and EDL muscles were removed from 8 week old control *mdx*/*S1PR3*^*+/−*^ and *mdx/S1PR3*^*−/−*^ males. No significant difference in weight was observed between the two genotypes, indicating no gross hypertrophy in the absence of S1PR3 (data not shown). Haematoxylin and eosin staining of cryosections revealed significantly less centronucleated myofibres in *mdx/S1PR3*^*−/−*^ compared to their control *mdx*/*S1PR3*^*+/−*^ littermates (Fig. 5A, quantified in Fig. 5B). There was also an increase in the size of both centronucleated and peripherally nucleated myofibres in *mdx/S1PR3*^*−/−*^ compared to their control *mdx*/*S1PR3*^*+/−*^ littermates (Fig. 5A, quantified in Fig. 5C and D).

To confirm the improvement in the phenotype of *mdx/S1PR3*^*−/−*^ mice compared to control *mdx*/*S1PR3*^*+/−*^ we also analysed the diaphragm, which is known to be particularly sensitive to the absence of dystrophin in *mdx* mice (Stedman et al., 1991). Again, the number of centronucleated fibres was lower in *mdx*/*S1PR3*^*−/−*^ compared to their control *mdx/S1PR3*^*+/−*^ littermates (Fig. 5E, quantified in Fig. 5F). There was also a general increase of the size of myofibres in the diaphragms of *mdx/S1PR3*^*−/−*^ compared to control *mdx*/*S1PR3*^*+/−*^ mice (Fig. 5E, quantified in Fig. 5G and H).

## Discussion

Muscle regeneration is compromised when S1P biosynthesis is inhibited (Nagata et al., 2006b), and S1P levels increase during muscle regeneration via both control of S1P biosynthesis and catabolism (Danieli-Betto et al., 2010, Loh et al., 2012). The main cells responsible for muscle regeneration, satellite cells, are normally mitotically quiescent but when needed, are recruited to produce myoblasts to perform muscle repair. S1P stimulates entry of activated satellite cell into the cell cycle and promotes proliferation (Calise et al., 2012, Loh et al., 2012, Nagata et al., 2006a, Nagata et al., 2006b).

Here we continued examination of S1P signalling in the control of satellite cell function by investigating the role of the S1PRs. Murine myoblasts express *S1PR1*, *S1PR2* and *S1PR3* in a dynamic fashion through myogenic progression. *S1PR1* was expressed at higher levels in differentiating myogenic cells compared to proliferating, while levels of *S1PR2* and *S1PR3* were unchanged through myogenic progression. Interestingly, *S1PR3* was highly expressed in C2 reserve cells and freshly isolated quiescent satellite cells, compared to the low levels when these cells were proliferating; results consistent with previous observations of high *S1PR3* expression in reserve cells (Rapizzi et al., 2008), and in quiescent satellite cells using microarrays (Pallafacchina et al., 2010) or by immunostaining (Danieli-Betto et al., 2010). *S1PR3* is at low levels in both satellite cells isolated from *mdx* mice and in growing mice compared to quiescent satellite cells from mature healthy muscle, likely reflecting their more active state during growth and under dystrophic conditions (Pallafacchina et al., 2010). Furthermore, a decrease of S1PR3 expression during the first week of regeneration has also been noted (Danieli-Betto et al., 2010). Although, Danieli-Betto and collaborators report increased levels of S1PR3 by western-blot of whole regenerating and neonatal muscle (Danieli-Betto et al., 2010), satellite cells are likely to contribute only a minor fraction of isolated protein. Our study clearly associates quiescent myogenic cells with high *S1PR3* expression, and much lower levels in the proliferative state.

To counter modulation of S1PR levels during myogenic progression, we used retroviral-mediated expression to examine the effects of maintaining high S1PR levels on satellite cell function. Constitutive expression of either S1PR1 or S1PR2 did not affect the already robust myoblast proliferation or differentiation stimulated by our culture conditions. S1PR2 is involved in mediating mitogenic and/or differentiation effects of S1P in myogenic cells (Calise et al., 2012, Germinario et al., 2012, Loh et al., 2012). Furthermore, delayed muscle regeneration is seen if S1PR2 function is inhibited using JTE-013 in wildtype mice, or in *S1PR2*^*−/−*^ mice (Germinario et al., 2012). In our experiments, we saw no effects of S1PR2 over-expression on proliferation, although it is likely that the cells are already proliferating at a high rate.

Over-expression of *S1PR3* suppressed cell cycle progression in satellite cell-derived myoblasts, and caused a small reduction of fusion into myobubes. Conversely, satellite cells isolated from *S1PR3*^*−/−*^ mice exhibited enhanced proliferation, with cells often still proliferating when wildtype cells were exiting the cell cycle and adopting a phenotype consistent with self-renewal (Zammit et al., 2004). However, rather than any overt modification in myogenic progression, the proportion of satellite cells expressing Pax7, MyoD and Myogenin was similar between wild type and *S1PR3*^*−/−*^-derived satellite cells, reinforcing the idea that an increase in proliferation is the main effect of the absence of S1PR3. Increased proliferation can also be associated with better differentiation in various cell types, including satellite cells (Abou-Khalil et al., 2009), and *S1PR3*-null satellite cells differentiated into myotubes more extensively. We tried to avoid any effect on fusion of the enhanced proliferation in satellite cells lacking S1PR3 by seeding them at high confluence immediately before switching to differentiation medium. However, the possibility that the increased differentiation was partly because of a higher cell density due to the greater proliferative ability of *S1PR3*-null cells cannot be excluded.

Although S1P is normally associated with pro-mitogenic effects in many cell types (An et al., 2000, Harada et al., 2004), administration of S1P can also inhibit cell division in proliferating C2 cells, which is prevented by inhibition of S1PR1 or S1PR2 (Donati et al., 2005, Rapizzi et al., 2008). Furthermore, S1PR2 can block PDGF-induced proliferation of murine embryonic fibroblasts and rat hepatocytes (Goparaju et al., 2005, Ikeda et al., 2003) and S1PR5 inhibits proliferation of human oesophageal cancer cells whether it binds S1P or not (Hu et al., 2010). Previous studies reveal that S1PR3 is associated with promoting proliferation in mesangial cells and myofibroblasts, and also in certain pathogenic conditions (An, 2000, Hsu et al., 2012, Katsuma et al., 2002, Wu et al., 2008), but, to our knowledge, there are no other reports of S1PR3 suppressing proliferation. Calise et al. demonstrated that S1P augments proliferation in serum-starved satellite cells, which is reduced by exposure to various small-molecule inhibitors, indicating that S1PR1-4 were all involved in the pro-mitogenic effects of S1P (Calise et al., 2012). However, as the authors acknowledge, the antagonists are not specific, with CAY10444 or BLM241 used to block S1PR3 having been reported to be non-specific (Jongsma et al., 2006). *SiRNA* against each S1PR revealed that only the knockdown of either *S1PR2* or *S1PR3* mRNA reduced S1P-induced proliferation (Calise et al., 2012). We speculate that the discrepancies between the two studies arise from a combination of factors including; examining satellite cells with transient, incomplete knockdown of *S1PR3* using *SiRNA* versus examining *S1PR3*-null satellite cells that have never expressed *S1PR3*; examining *siRNA-*transfected satellite cells after serum-starvation (for up to 22 h) and then in the presence of 1mM SIP for another 20 h compared to examining *S1PR3*-null satellite cells in standard high-serum proliferation medium; potential off-target effects of the *siRNA* versus germline-deletion of *S1PR3*. It would be interesting to determine the effects of *siRNA*-mediated *S1PR3* knockdown in satellite cells cultured in standard high-serum proliferation medium. It is also possible that S1PR3 may act independently of S1P binding as demonstrated recently for S1PR1 in endothelial cells in response to biomechanical signals (Jung et al., 2012) and that there might be cross-talk in signalling pathways with other receptors types, such as IGF, PDGF and TGF-β (Donati et al., 2013, Liu et al., 2010).

Importantly, assessing satellite cell function in vivo showed that in the absence of S1PR3, acute regeneration was enhanced after a single insult with bigger myofibre present, with this effect more pronounced after several rounds of injury. We also examined chronic muscle regeneration using the *mdx* mouse model of Duchenne muscular dystrophy, which undergoes continuous cycles of muscle degeneration and regeneration (De la Porte et al., 1999). *Mdx* mice lacking *S1PR3* had an improved dystrophic phenotype compared to their *mdx* littermates, with fewer myofibres with central nucleation (a hallmark of myofibre regeneration) and a larger mean myofibre cross-sectional area in both Tibialis Anterior and diaphragm (a severely affected muscle in *mdx* mice—(Stedman et al., 1991)). Increased satellite cell proliferation in the absence of S1PR3 would presumably generate more myoblasts allowing muscle repair to be enhanced during acute muscle regeneration, producing bigger myofibres. However, increased satellite cell proliferation in the *mdx* model could reduce signs of trauma (myofibres with centrally-located myonuclei) since the ongoing repair of damaged fibres may be more efficient, leading to less need for complete myofibre regeneration. This would also result in an increased mean myofibre size in *mdx/S1PR3-*null mice compared to controls, as we observed. Interestingly, Loh and colleagues recently showed in *mdx* muscle that S1P levels are low and S1P catabolism is increased. The dystrophic muscle phenotype in *mdx* mice was improved via pharmacological inhibition of sphingosine phosphate lyase through an S1PR2-dependent mechanism involving suppression of Rac1 (Loh et al., 2012). Elevation of intracellular S1P levels also suppresses muscle wasting in flies with dystrophic muscle, showing the conservation of this signalling pathway (Pantoja et al., 2013).

*S1PR3*^*−/−*^ is a germline knockout, so S1PR3 is absent in all cell types, including inflammatory cells and endothelial-associated pericytes/mesoangioblasts, which could also contribute to enhanced muscle regeneration. The initial characterization of the *S1PR3* knockout mice did not show any change in the immune cell number however (Ishii et al., 2001). Cell types from the microvasculature wall such as pericytes/mesoangioblasts have myogenic potential and are regulated by S1P, but S1P exerts its pro-mitogenic effects through S1PR2, although S1PR3 is highly expressed (Donati et al., 2007). Therefore, mesoangioblast function could be affected by loss of S1PR3 signalling in *S1PR3*-null mice. S1PR3 is also involved in endothelial cell barrier integrity, though its exact role remains controversial. A more disrupted barrier in *S1PR3*-null mice could allow more pericytes/mesoangioblasts to be released to participate in muscle regeneration, although several publications mention that S1PR3 activation, rather than removal, may increase barrier disruption (e.g.-Singleton et al., 2007). However, the recent evidence that satellite cells are responsible for muscle regeneration (e.g. Lepper et al., 2011 reviewed in Relaix and Zammit, 2012), combined with the clear effects of lack of S1PR3 on satellite cell proliferation ex-vivo, indicates that this is likely to be the major factor in the improved muscle repair that we observe in *S1PR3*-null mice.

Having demonstrated a role of S1PR3 in satellite cell function and myogenesis, the next challenge is to decipher the signalling pathways downstream of this receptor. The sphingosine-1-phosphate receptors belong to the GPCR family and so operate through different types of G-proteins, which in turn, control multiple signalling pathways involved in cell survival, migration and proliferation (Donati et al., 2013, Spiegel and Milstien, 2003). Several candidates have been associated with the role of S1PR3 in proliferation in other cell types (Rosen et al., 2009) but which pathways act to mediate the effects of S1PR3 in satellite cells is unclear. Smad3 is involved in S1PR3-mediated myofibroblast proliferation and differentiation (Keller et al., 2007), and recent studies have elucidated the role of smad3 in satellite cell function (Ge et al., 2011, Ono et al., 2011). Smad3 is a key player in the TGF-β mediated repression of myogenesis (Liu et al., 2001) and different studies have also described overlapping signalling between S1P and TGF-β (Liu et al., 2010, Xin et al., 2004). Another candidate is Erk, the phosphorylation of which can be regulated by S1P signalling in different cell types and is associated with proliferation (Rodgers et al., 2009). In C2C12, Erk has a bimodal function, being necessary both for maintaining cells in an undifferentiated state, and for the hypertrophy of myotubes (Wu et al., 2000).

A picture is emerging whereby signalling through S1PR3 may contribute to control of satellite cell quiescence, while signalling operating through S1PR2 may then drive S1P-mediated proliferation and/or differentiation. Satellite cells are actively maintained in a quiescent state (Dhawan and Rando, 2005, Montarras et al., 2013). For example, the calcitonin receptor is highly expressed in quiescent satellite cells, and signals to attenuate the entry of satellite cells into the cell cycle, since addition of calcitonin blocks incorporation of BrdU (Fukada et al., 2007). Similarly, the Tie2 receptor and its ligand angiopoietin, are involved in satellite cell self-renewal, requiring re-entry into quiescence (Abou-Khalil et al., 2009). More recently, Notch signalling has also been demonstrated to be important for maintaining satellite cell quiescence, since when it is inactivated by knocking out RBP-J, satellite cells spontaneously activate and differentiate without self-renewing, leading to depletion of the satellite cell pool (Bjornson et al., 2011, Mourikis et al., 2011). Analysis of S1PR3 expression and function in other cells types will illuminate if S1PR3 is a global regulator of quiescence.

In conclusion, we have shown that S1PR3 is highly expressed in quiescent myoblasts and falls as they activate and enter the cell cycle. If expression of S1PR3 is maintained in proliferating cells, this then leads to slower progression through the cell cycle, while lack of signalling through S1PR3 enhances proliferation. The absence of S1PR3 enhances both acute and chronic muscle regeneration in vivo. Therefore, S1PR3 has a key role in controlling satellite cell function, being amongst a cohort of receptors regulating satellite cell quiescence.

## Figures and Tables

**Fig. 1:**
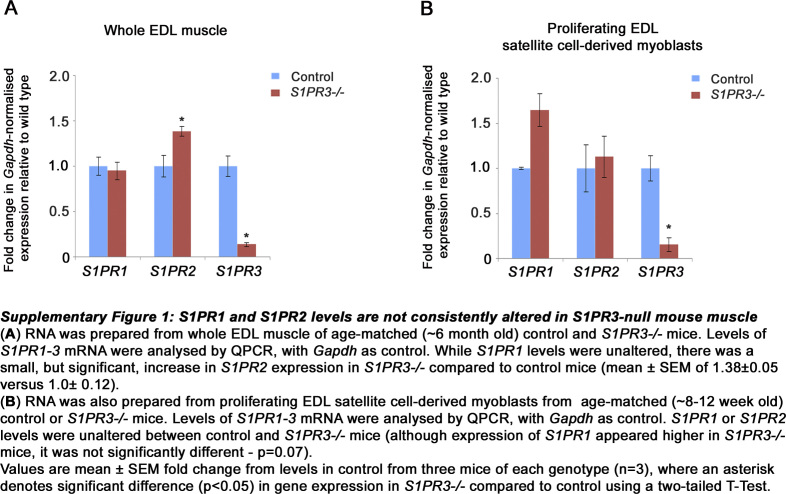
S1PR1 and S1PR2 levels are not consistently altered in S1PR3-null mouse muscle (A) RNA was prepared from whole EDL muscle of age-matched (~6 month old) control and *S1PR3*^*−/−*^ mice. Levels of *S1PR1-3* mRNA were analysed by QPCR, with *Gapdh* as control. While *S1PR1* levels were unaltered, there was a small, but significant, increase in *S1PR2* expression in *S1PR3*^*−/−*^ compared to control mice (mean±SEM of 1.38±0.05 versus 1.0±0.12). (B) RNA was also prepared from proliferating EDL satellite cell-derived myoblasts from age-matched (~8–12 week old) control or *S1PR3*^*−/−*^ mice. Levels of *S1PR1-3* mRNA were analysed by QPCR, with *Gapdh* as control. *S1PR1* or *S1PR2* levels were unaltered between control and *S1PR3*^*−/−*^ mice (although expression of *S1PR1* appeared higher in *S1PR3*^*−/−*^ mice, it was not significantly different—*p*=0.07). Values are mean±SEM fold change from levels in control from three mice of each genotype (*n*=3), where an asterisk denotes significant difference (*p*<0.05) in gene expression in *S1PR3*^*−/−*^ compared to control using a two-tailed *T*-test.

## Footnotes

Appendix A Supplementary data associated with this article can be found in the online version at 10.1016/j.ydbio.2013.07.006.
